# Narcissism and suicide risk

**DOI:** 10.1186/s12991-022-00380-8

**Published:** 2022-01-22

**Authors:** Glen O. Gabbard

**Affiliations:** Clinical Professor of Psychiatry, Baylor College of Medicine, Houston, TX USA

**Keywords:** Narcissism, Countertransference, Transference, Shame and mentalization

## Abstract

We all have narcissism, but in some cases, the perception of narcissism becomes extreme and pathological. Systematic research has shown that there are three subtypes typical of narcissistic personality disorder: the grandiose/oblivious, the vulnerable/hypervigilant, and the high-functioning subtype. Both biological and psychological factors are at work, but the true cause of pathological narcissism has not been established. The psychotherapy of narcissistic personality disorder (NPD) is complicated and often frustrating because of the difficulty in engaging a person with narcissistic personality disorder in a psychotherapeutic process. Suicide risk is not rare in patients with narcissism, particularly in the context of severe narcissistic injury, where the patient feels shamed and/or vilified. In conclusion, narcissistic patients are difficult to treat, but the risk of suicide makes it imperative for clinicians to stay involved in the treatment and assist the patient in understanding their vulnerabilities.

## Background

The background of this article is to underscore the risks of suicide and psychological decompensation in patients with narcissistic personality disorder. The existing literature suggests that some narcissistic patients will die by suicide but others are capable of improving their vulnerability with the help of a long-term psychotherapy geared to enhancing their self-esteem and increasing their capacity to mentalize the internal processes of the patient. Recent publications have suggested that there are both biological and psychological factors in the development of the condition.

The diagnosis of narcissistic personality disorder has been difficult and controversial for many years. The usual characteristics of this disorder include a need for admiration from others, a grandiose manner of relating, and a lack of empathy for others. Some will require a great deal of admiration from others, while some individuals with narcissistic pathology will keep to themselves and have very little interest in what others are doing or thinking.

In recent years, more systematic information has been available to understand the nature of narcissistic personality disorder. Russ et al. [1] used the Shedler–Westen Assessment Procedure-II (SWAP-II) to study how clinicians make a diagnosis of narcissistic personality disorder (NPD). 1200 psychiatrists and clinical psychologists participated and used the SWAP-II to characterize most typical patients of NPD. Hence the prototypes that were gathered were based on clinical perspectives. From this exercise, a total of 255 patients met DSM-IV criteria for NPD. Q-factor analysis was used to identify three subtypes, which the authors labelled as grandiose-malignant, fragile, and high-functioning. They also found that core features of NPD include interpersonal vulnerability, difficulty regulating affect, competitiveness, and underlying emotional distress, all of which are absent from the DSM-V criteria.

The grandiose/oblivious subtype was characterized by exaggerated self-importance, lack of remorse, interpersonal manipulativeness, seething anger, pursuit of interpersonal power, and pursuit of privilege. Persons in the vulnerable/hypervigilant subtype were characterized by an exquisite sensitivity to what others are saying, a tuning in to every comment that might be critical, a shyness to the point where NPD could be confused with Avoidant PD, a proneness to shame and hurt feelings and a masochistic conviction that they have suffered more than anyone. The high-functioning subtype may be difficult to diagnose as the person may appear congenial, outgoing, energetic, and articulate. However, with careful assessment, they are noted to be individuals with an exaggerated sense of self-importance, a capacity to be charming and likable, an appearance of one who is listening and valuing psychotherapy, a lack of narrative continuity, a false self-structure, and a tendency to repeat themselves when interviewed.

In clinical practice, it is important to recognize that the subtypes are not etched in granite. A grandiose narcissist can collapse into a vulnerable narcissist when shamed or criticized. Other patients may have features of all three of the subtypes. Another factor that contributes to the pleomorphism is co-morbidity. One regularly discovers that a great many narcissistic individuals have other characterological tendencies as well—obsessive–compulsive issues, antisocial tendencies, borderline elements, and masochistic propensities.

In fact, in an impressive inquiry, Lee et al. [2] studied 68 normal controls, 29 psychiatric controls, and 98 personality-disordered subjects (*n* = 195). The investigators found that higher levels of the oxidative stress marker 8-OH-DG are associated both with the diagnosis of narcissistic personality disorder and borderline personality disorder. One can infer that there may be a neurological factor that could assist us in understanding the close relationship between NPD and BPD. In addition, the research of Lee and colleagues suggests that NPD appears to be located somewhere on a spectrum that includes the callousness of antisocial personality disorder, on one hand, and the interpersonal hypersensitivity of borderline personality disorder, on the other. The biological data would suggest that NPD and BPD share interpersonal hypersensitivity but do different things with this experience at the behavioral level. They react in somewhat different ways, but perhaps share an underlying trigger. Clinicians may be surprised by the emergence of antisocial features on this spectrum. However, one must keep in mind that dishonesty can stem from the desperate need to impress others. This is not a rare occurrence.

A body of clinical research has started to emerge that helps us understand the inner workings of the narcissist’s mind. Caligor et al. [3] found that individuals with NPD have a need for approval and react with hostility when their expectations are not met. De Panfilis et al. [4] have studied facial emotional recognition and social cognitive correlates of narcissistic features. These investigators found that NPD is associated with both hypersensitivity to social feedback and seeming indifference to social feedback. In their study of 200 non-clinical individuals who self-reported NPD features, those with higher NPD features were faster at recognizing neutral and low-intensity emotional stimuli. This response pattern mediated the association between NPD features and increased anger about rejection. Hence, individuals with high NPD traits are hypervigilant to subtle negative emotions and neutral expressions in the faces of others. This increased sensitivity for negative and neutral facial cues, however, does not seem to translate into greater empathic ability and greater propensity to take others’ needs and perspectives into account. Baskin-Sommers [5] points out that the lack of empathy for others results from poor motivation to consider others’ feelings, not from an inability to recognize the feelings of others. Some may be capable of mentalizing what is going on with others but do not want to be bothered by it.

One of the common findings with NPD patients is that they may not speak in a way that implies a dialogue. The observer will note that the lilt of the speech tends to go up rather than down, as if there will not be an opening for anyone else to speak. Therapists who try to break in and offer an opinion will often find that the patient will stop talking, break eye contact, and appear irritated since he or she has little interest in what the therapist is saying. Omnipotent control is central for the NPD patient. Hence, one can expect to be treated like a sounding board.

## Common countertransference

Therapists may need to pace themselves. It is common for therapists to feel that they are being talked “at” rather than “to”. They may feel like they are not actually part of the conversation. Rather, it is as though they are an onlooker of a private conversation from which they are somehow excluded. They may feel like an unimportant observer who resents the fact that he/she is not the primary object for the patient. One of the common reactions in narcissistic patients is that they do not appear to respond to what the therapist says. They may not laugh at jokes. They rarely agree with what the therapist has said. They may take the therapist’s comments in another direction. They may look at their watch while the therapist is talking to see how much time they have left so they can finish. They may check their phone for messages until they have an opening. They rarely provide a positive facial reaction that might express empathy with what the therapist is saying. The early attachment problems found so commonly in NPD patients often lead to a deficit in the capacity to empathize.

So what does the therapist do with this no-response? A major goal is to stabilize the sense of self, recognizing that a lack of secure attachment in childhood made it difficult for these patients to find themselves in the eyes of their caregivers or parents. Therapists need to allow the patient to draw them into their internal world. In this regard, the therapist accepts what is projected into her while also maintaining a clear view of her own state of mind while taking note of the process. The therapist may be able to assist the patient in thinking more broadly about the possibility of multiple perspectives on whatever situation is being discussed. One should remember a consistent finding in psychotherapy research, i.e., the most powerful predictor of outcome in psychotherapy is the therapeutic alliance. Hence, the therapist needs to immerse herself in what the patient is saying and resist the descent into contempt that is so common for all of us.

## Suicide risk in narcissistic individuals

Suicidal wishes are not rare in those with narcissistic personality disorder. However, the factors contributing to suicide among narcissistic individuals are not well understood. Paul Links [6] has pointed out that with patients on the narcissistic spectrum, the wish to kill oneself can emerge without the presence of a depressed state. Indeed, suicidal feelings may grow out of a desperate need to regulate self-esteem or protect a pathological self-image of perfection. In addition, an acute narcissistic injury can produce intense shame to the point where suicide seems like the only option. Whereas guilty individuals may feel they are not living up to a standard, shame-prone patients, such as vulnerable narcissists, have the sense of being irreparably defective.

Shame is sometimes defined as a sense of falling short of what one should be. Shame is often at the core of narcissistic individuals, who struggle with an acute sense of having failed in many aspects of their lives. They feel certain that others are thinking badly about them. When “caught” by someone watching them, a sense of painful humiliation may emerge. Seeing and being seen are central to narcissistic patients. They may feel that their secret sense of being “phony” or “fake” is now exposed. Suicide may feel like the only way out, especially after a devastating humiliation.

It is not well recognized, but suicidal behavior is a clinically significant but underestimated cause of mortality in narcissistic personality disorder. In a sample of 446 suicide attempters, patients with cluster B personality disorder diagnoses (*n* = 254) were compared in terms of expected lethality and impulsivity. This rigorous investigation showed that subjects with NPD were less impulsive but had higher lethality than those without NPD [7].

A number of treatment considerations are relevant to the outcome with narcissistic patients who are feeling shame, defeat, self-loathing, and other intense reactions. The therapist must emphasize the importance of honesty in working with the patient. Whether or not the patient can remain an outpatient needs to be discussed openly and continuously so that the risk of suicide must be part of the regular discourse. It is useful after hearing a patient say that suicide is not a risk to ask again, “Are you really telling me the truth?” A surprising number of patients will respond to this query with more truthfulness the second time it is asked.

Another key point is that there is a difference between treatment and management of the suicidal patient. Management involves removing sharp objects, continuous observation in a hospital, and even restraints in some situations. Treatment, on the other hand, involves altering the wish to die. It requires a therapeutic effort to discover the patient’s fantasies associated with suicide and the evolution of self-loathing.

The patient my wish for the therapist to be a godlike figure, even a savior, while also wishing to destroy the therapist and ruin his or her reputation. It is common for narcissistic patients to disparage the therapist’s competence by accusing him or her of being inattentive or oblivious to their lethality. Patients may actually expect the therapist to be omniscient.

Countertransference must be repeatedly be considered in the process. The therapist may be tormented by a sadistic patient who is filled with hate and anger. Suicidal patients may be particularly bitter about how the treatment is going and let the therapist know that they are getting poor treatment. One associated risk is that the patient may view the therapist as a target of contempt, because the therapist is not moving fast enough or not honoring the wishes of the patient (who may be demanding discharge from the hospital). If this conflict continues, some therapists may unconsciously contribute to a suicide attempt by neglecting the patient or “forgetting” to see the patient. The experience of being devalued relentlessly may lead them to act in ways that are significantly different from their usual mode of practice. Because of this vulnerability that is often associated with the treatment of narcissistic patients, practitioners should have a consultant to whom they can speak periodically when they feel overwhelmed, devalued, or ignored by their patient.

## Conclusions

Patients with narcissistic personality disorder are often thought to be less likely to make the kind of random non-lethal suicide attempts typical of the impulsivity associated with attempts made by patients with borderline personality disorder. However, Ronningstam et al. [8] point out that NPD patients are at high risk for completed suicides or highly lethal attempts without warning signs or self-disclosures. Indeed, they are made with the intention to end their lives. Therefore, professionals involved in the treatment must assume that an impulsive behavior designed to end their lives is always a possibility.

## Data Availability

Availability of data and materials came from established journals in the field of personality disorders. No clinical or research data were used. The article is an overview of what is known about narcissistic personality disorder and suicide risk. Data sharing is not applicable as no data sets were generated or analyzed.

## Abbreviation

NPD: Narcissistic personality disorder

## References

[1] Russ E, Shedler J, Bradley R, Westen D (2008). Refining the construct of narcissistic personality disorder: diagnostic criteria and subtypes. Am J Psychiatry.

[2] Lee RJ, Gozal D, Coccaro EF, Fanning J (2020). Narcissistic and borderline personality disorders: relationship with oxidative stress. J Pers Disord.

[3] Caligor E, Levy KN, Yeomans FE (2015). Narcissistic personality disorder: diagnostic and clinical challenges. Am J Psychiatry.

[4] De Panfilis C, Antonucci C, Meehan KB, Cain NM, Soliani A, Marchesi C (2019). Facial emotion recognition and social-cognitive correlates of narcissistic features. J Pers Disord.

[5] Baskin-Sommers A, Krusemark E, Ronningstam E (2014). Empathy in narcissistic personality disorder: from clinical and empirical perspectives. Pers Disord.

[6] Links PS, Ogrodniczuk JS (2013). Pathological narcissism and the risk of suicide. Understanding and treating pathological narcissism.

[7] Blasco-Fontecilla H, Baca-Garcia E, Dervic K, Perez-Rodriguez MM, Lopez-Castroman J, Saiz-Ruiz J (2009). Specific features of suicidal behavior in patients with narcissistic personality disorder. J Clin Psychiatry.

[8] Ronningstam E, Weinberg I, Goldblatt M, Schechter M, Herbstman B (2018). Suicide and self-regulation in narcissistic personality disorder. Psychodyn Psychiatry.

